# Application of Individualized Speed Thresholds to Interpret Position Specific Running Demands in Elite Professional Rugby Union: A GPS Study

**DOI:** 10.1371/journal.pone.0133410

**Published:** 2015-07-24

**Authors:** Cillian Reardon, Daniel P. Tobin, Eamonn Delahunt

**Affiliations:** 1 Leinster Rugby, Dublin, Ireland; 2 School of Public Health, Physiotherapy and Population Science, University College Dublin, Dublin, Ireland; 3 Institute for Sport and Health, University College Dublin, Dublin, Ireland; University of Rome Foro Italico, ITALY

## Abstract

A number of studies have used GPS technology to categorise rugby union locomotive demands. However, the utility of the results of these studies is confounded by small sample sizes, sub-elite player status and the global application of absolute speed thresholds to all player positions. Furthermore, many of these studies have used GPS units with low sampling frequencies. The aim of the present study was to compare and contrast the high speed running (HSR) demands of professional rugby union when utilizing micro-technology units sampling at 10 Hz and applying relative or individualised speed zones. The results of this study indicate that application of individualised speed zones results in a significant shift in the interpretation of the HSR demands of both forwards and backs and positional sub-categories therein. When considering the use of an absolute in comparison to an individualised HSR threshold, there was a significant underestimation for forwards of HSR distance (HSRD) (absolute = 269 ± 172.02, individualised = 354.72 ± 99.22, p < 0.001), HSR% (absolute = 5.15 ± 3.18, individualised = 7.06 ± 2.48, p < 0.001) and HSR efforts (HSRE) (absolute = 18.81 ± 12.25; individualised = 24.78 ± 8.30, p < 0.001). In contrast, there was a significant overestimation of the same HSR metrics for backs with the use of an absolute threshold (HSRD absolute = 697.79 ± 198.11, individualised = 570.02 ± 171.14, p < 0.001; HSR% absolute = 10.85 ± 2.82, individualised = 8.95 ± 2.76, p < 0.001; HSRE absolute = 41.55 ± 11.25; individualised = 34.54 ± 9.24, p < 0.001). This under- or overestimation associated with an absolute speed zone applies to varying degrees across the ten positional sub-categories analyzed and also to individuals within the same positional sub-category. The results of the present study indicated that although use of an individulised HSR threshold improves the interpretation of the HSR demands on a positional basis, inter-individual variability in maximum velocity within positional sub-categories means that players need to be considered on an individual basis to accurately gauge the HSR demands of rugby union.

## Introduction

Rugby union game play is characterized by high intensity intermittent activity, whereby periods of high intensity static and locomotive activity are interspersed with periods of lower intensity aerobic activity and rest [1–8]. A review of existing research reveals that the progression of the professional era has been accompanied by a chronological trend towards an increase in the intensity of game play, and consequently the physical fitness requirements of players [7].

Previous research has reported on the different locomotive activity profiles of players within the forward and back units along with their respective positional subcategories [1–10]. Roberts et al. [8] reported that forwards spend three to four-fold the amount of time in intense static activities (e.g. scrums, rucks and mauls) in comparison to backs; which is directly attributable to the specific role played by forwards in the set-piece and breakdown elements of the game. Traditionally in the published literature it has been reported that backs cover significantly more total distance at high speed than forwards due to the specific demands of their role in game play, combined with their greater opportunity for open-field running [3–6,10]. However, contradictory evidence exists, with Austin and colleagues [7] reporting similar total distances covered at sprint speed by back row forwards and outside backs (547 ± 55 m and 558 ± 282 m respectively). More recently Cahill et al. [9] observed that forwards cover slightly more total distance at high speed in comparison to backs.

Some of the disparity in the aforementioned research may be due to differences in the methodologies utilized for the categorisation of speed zones during game play. Earlier research used time motion analysis to subjectively describe locomotion during match play [8,10]. Although acceptable inter- and intra-rater reliability has been reported for this method, no validation against quantitative data exists to verify the accuracy of this type of movement categorisation.

A number of studies have used GPS technology to categorise rugby union locomotive demands [3,9,11,12]. However, the utility of the results of these studies are confounded by either a small player sample size [3,11,12] or a small game sample size [3,11,12]. Making general recommendations on the locomotive game demands of rugby union considering the above limitations may be erroneous. Furthermore, all of the four aforementioned studies used GPS technology operating at a relatively low sampling rate (rate ≤ 5Hz). Newer, 10Hz GPS units have been reported to be up to six-fold more reliable than 5 Hz systems for the measurement and quantification of instantaneous velocity [13]. As such, further research is required to quantify the locomotive demands of professional rugby union players using higher frequency GPS units.

Another significant methodological issue of note is the adoption and utilization of default or absolute speed zones in comparison to relative or individualised speed zones. Despite the fact that Duthie et al. [14] reported the maximum velocity (Vmax) of forwards to be 37% lower than that of backs, recent studies have persisted in reporting locomotive demands relative to arbitrary, pre-determined speed zones [11,12]. Consequently, the adoption of this approach is likely to result in the over- or underestimation of the high speed running demands of rugby union players. Suarez-Arrones et al. [12] reported a significantly greater total high speed running distance for inside backs and centres (86 ± 39 m and 232 ± 37 m respectively) than for front and back row forwards (635 ± 47 and 292 ± 44 respectively) when using an absolute lower velocity threshold of 5.5 m·s^-1^ (20 km·h^-1^) to determine high speed running distance. In contrast, Cahill et al. [9] reported no difference in total high speed running distance covered by forwards (897 m) and backs (872 m) when an individualised threshold of 51% Vmax was used. This demonstrates the potential for discrepancy when employing individualised versus absolute speed zones to determine high speed running demands, which has significant implications when designing sport specific conditioning protocols.

The purpose of this study was to compare and contrast the high speed running demands of professional rugby union when utilizing relative or individualised (IND) speed zones versus the absolute (ABS) default settings of the GPS manufacturer. It is the present authors’ contention that the utilization of GPS technology sampling at 10 Hz, combined with reporting on a large sample of players (and specific positional unit sub-categories) and games, with the inclusion of individualised speed zones will garner more applicable and reliable data on the game related high speed running demands of professional rugby union.

## Methods

### Participants

Thirty six elite professional players from a RaboDirect Pro12 team volunteered to participate in the study. The study was approved by the University College Dublin Human Research Ethics Committee (LS-14-03-Delahunt). Furthermore, each participant signed an informed consent form approved by the University College Dublin Human Research Ethics Committee. The participants (age 27.2 ± 3.9 years, body mass 99.2 ± 24.4 kg, height 1.85 ± 0.43 m) cumulatively provided 193 GPS files from 20 games in the RaboDirect Pro12 league. Each player provided at least one GPS file with the largest number of files provided by any one player being twelve.

### Procedures

All matches took place between September 2013 and May 2014 on a Friday, Saturday or Sunday and were played on 12 different grounds used by clubs participating in the RaboDirect Pro12. Each consenting player wore a GPS micro-technology unit (mass = 67 g, size = 50·90 mm) (10 Hz V5.0 and 10 Hz S5, Catapult Innovations, Scoresby, VIC, Australia) in a bespoke pocket fitted in his playing jersey on the upper thoracic spine between the scapulae. The GPS device captured data at a sampling frequency of 10 Hz. The reliability of the unit has previously been demonstrated as acceptable for measuring speed and distances in team sports [13,15–17]. The 10 Hz sampling frequency has been reported to be up to six times more reliable at measuring instantaneous velocity than 5 Hz units [13]. All participants were familiarized with the devices as part of their day to day training and playing practices. Each player wore the same assigned GPS unit throughout the course of the data collection period.

The GPS units were switched on least 10 minutes prior to the game to ensure a full high quality satellite signal. Following the game, GPS data was downloaded to a laptop and analyzed with Sprint 5.1 software (Catapult Innovations, Scoresby, VIC, Australia). Real time data was analyzed and appropriate periods and substitutions noted in the software, enabling knowledge of duration of each player’s participation in the game. The raw data files were later exported from Sprint 5.1 software into Microsoft Excel (Microsoft Corporation, USA) and subsequently PASW (version 18) for statistical analysis. Analysis of substitution times was conducted to determine the average duration of match participation for each position. GPS files were only included in statistical analysis if a player had participated for at least the average duration for his position.

For the purposes of data analysis and comparisons with previous studies [5], players were broadly grouped into Backs or Forwards. Secondly, they were assigned to a sub-category of positions of which there were ten. These positional groups have previously been reported to have distinctive game demands [5]. The positional sub-categories used were as follows: (1) Prop; (2) Hooker; (3) 2^nd^ Row; (4) Number 8; (5) Flanker; (6) Out-half; (7) Scrum-half; (8) Centre; (9) Wing; (10) Full-back.

#### Locomotor Variables

The total distance (m) and total distance relative to playing time (m·min^-1^) was calculated for each data file. The maximum velocity (Vmax) of each participant was established at the end of the data collection period from analyzing all training and playing data throughout the season. This included dedicated speed training sessions. The relative or individualized speed zones were retrospectively applied to all game data with knowledge of maximum velocity achieved for each participant during the season. The arbitrary lower threshold of the high speed running (HSR) zone used by the GPS supplier to classify HSR was 5m·s^-1^, which has been used in classifying high intensity running in GPS based rugby league studies [18]. Therefore the distance travelled at a velocity above 5 m·s^-1^ was summed in the report to represent a figure for absolute high speed running for each participant. In order to calculate an individual percentage of maximum velocity to classify the individual lower threshold for high speed running (HSR), the arbitrary 5 m·s^-1^ was applied to the mean Vmax of the participants (8.3m·s^-1^). Therefore the individualized HSR lower threshold was calculated as follows: arbitrary threshold for HSR/mean of the group for Vmax

$$\frac{5\;\text{m}\cdot {\text{s}}^{-1}}{8.3\;\text{m}\cdot {\text{s}}^{-1}\;}=0.60$$

As such the individual or relative lower threshold for HSR was set at 60% Vmax. The individual values for HSR (metres and % of total distance covered), were calculated for each participant and compared to the HSR meterage values derived from the arbitrary speed zones. The average speed of game play for each positional group (m·s^-1^) was also calculated. Each individual participant’s average speed in game play was also related to his Vmax to generate a percentage value for relative average game speed (%Vmax).

#### Statistical Analysis

A multivariate analysis of variance was performed to investigate differences in ABS and IND HSR demands of forwards. Three dependent variables were used: (1) HSR distance (HSRD); (2) % of total distance at HSR (HSR%); (3) number of HSR efforts (HSRE). The independent variable was the forward position unit.

A multivariate analysis of variance was performed to investigate differences in ABS and IND HSR demands of backs. Three dependent variables were used: (1) HSR distance (HSRD); (2) % of total distance at HSR (HSR%); (3) number of HSR efforts (HSRE). The independent variable was back position unit.

A multivariate analysis of variance of covariance was performed to investigate differences in forwards and back ABS HSR demands. Five dependent variables were used: (1) HSR distance (HSRD); (2) % of total distance at HSR (HSR%); (3) number of HSR efforts (HSRE); (4) HSR distance per minute (HSRDpm); (5) HSR efforts per minute (HSREpm). The independent variable was position (forward vs back). The covariate was the duration of time on the pitch.

A multivariate analysis of covariance was performed to investigate differences in forwards and back IND HSR demands. Five dependent variables were used: (1) HSR distance (HSRD); (2) % of total distance at HSR (HSR%); (3) number of HSR efforts (HSRE); (4) HSR distance per minute (HSRDpm); (5) HSR efforts per minute (HSREpm). The independent variable was position (forward vs back). The covariate was the duration of time on the pitch.

A multivariate analysis of covariance was performed to investigate differences between forwards and backs for the three dependent variables: total distance, relative distance and in-game maximum velocity. The independent variable was position (forward vs back).

For each position sub-category within the forward and back position units a separate multivariate analysis of variance was performed to investigate differences in ABS and IND HSR demands. Three dependent variables were used: (1) HSR distance (HSRD); (2) % of total distance at HSR (HSR%); (3) number of HSR efforts (HSRE). The independent variable was position sub-category.

Furthermore, descriptive data relative to the inter-individual differences in HSR output within positional sub-categories were also calculated.

All statistical analyses were performed with IBM SPSS Statistics 20 (IBM Ireland Ltd, Dublin, Ireland).

## Results

Concerning the forward position unit there was a statistically significant difference between ABS and IND HSR demands on the combined dependent variables, F (3, 206) = 8.00, p < 0.001, Wilk’s Lambda = 0.89, partial eta squared = 0.10. When the results of the dependent variables were considered separately, all variables reached statistical significance (Table 1).

**Table 1:** ABS vs IND HSR demands of forwards and backs.

Position	Variable	ABS	IND	Mean Difference	95% CI of Mean Difference	p Value	Effect size (partial eta squared)
Forward	HSRD (m)	269 ± 172.02	354.72 ± 99.22	-85.02	-123.23 – -46.82	< 0.001	0.08
	HSR% (%)	5.15 ± 3.18	7.06 ± 2.48	-1.90	-2.68 – -1.12	< 0.001	0.10
	HSRE	18.81 ± 12.25	24.78 ± 8.30	-5.96	-8.81 – -3.11	< 0.001	0.07
Back	HSRD (m)	697.79 ± 198.11	570.02 ± 171.14	127.76	72.03–183.49	< 0.001	0.10
	HSR% (%)	10.85 ± 2.82	8.95 ± 2.76	1.90	1.06–2.74	< 0.001	0.10
	HSRE	41.55 ± 11.25	34.54 ± 9.24	7.01	3.91–10.11	< 0.001	0.10

ABS = absolute high speed running threshold (5 m·s^-1^); IND = individual high speed running threshold (60% vMAX); HSR = high speed running; HSRD = high speed running distance; HSR% = % of total distance at HSR; HSRE = number of HSR efforts.

Concerning the back position unit there was a statistically significant difference between ABS and IND HSR demands on the combined dependent variables, F (3, 168) = 8.19, p < 0.01, Wilk’s Lambda = 0.87, partial eta squared = 0.12. When the results of the dependent variables were considered separately, all variables reached statistical significance (Table 1).

There was a statistically significant difference between forward and back ABS HSR demands on the combined dependent variables, F (5, 184) = 43.53, p < 0.001, Wilk’s Lambda = 0.45, partial eta squared = 0.54. When the results of the dependent variables were considered separately, all variables reached statistical significance (Table 2).

**Table 2:** Forward vs back ABS HSR demands.

Variable	Forward	Back	Mean Difference	95% CI of Mean Difference	p Value	Effect size (partial eta squared)
HSRD (m)	290.35 ± 180.03	672.56 ± 181.20	-382.21	-435.75 – -328.66	< 0.001	0.51
HSR% (%)	5.27 ± 3.07	10.71 ± 3.06	-5.44	-6.36 – -4.51	< 0.001	0.41
HSRE	20.06 ± 11.57	40.03 ± 11.68	-19.96	-23.42 – -16.50	< 0.001	0.40
HSRDpm (m·min^-1^)	3.77 ± 2.25	8.67 ± 2.31	-4.90	-5.59 – -4.20	< 0.001	0.50
HSREpm	0.26 ± 0.10	0.51 ± 0.09	-0.25	-0.30 – -0.20	< 0.001	0.39

ABS = absolute high speed running threshold (5 m·s^-1^); HSR = high speed running; HSRD = high speed running distance; HSR% = % of total distance at HSR; HSRE = number of HSR efforts; HSRDpm = high speed running distance per minute; HSREpm = high speed running efforts per minute.

There was a statistically significant difference between forward and back IND HSR demands on the combined dependent variables, F (5, 184) = 22.87, p < 0.001, Wilk’s Lambda = 0.61, partial eta squared = 0.38. When the results of the dependent variables were considered separately, all variables reached statistical significance (Table 3).

**Table 3:** Forward vs back IND HSR demands.

Variable	Forward	Back	Mean Difference	95% CI of Mean Difference	p Value	Effect size (partial eta squared)
HSRD (m)	359.09 ± 140.38	564.68 ± 141.32	-205.58	-247.35 – -163.81	< 0.001	0.33
HSR% (%)	6.79 ± 2.56	9.28 ± 2.50	-2.49	-3.26 – -1.73	< 0.001	0.18
HSRE	25.16 ± 8.91	34.07 ± 8.99	-8.91	-11.57 – -6.24	< 0.001	0.18
HSRDpm (m·min^-1^)	4.77 ± 1.74	7.41 ± 1.76	-2.63	-3.18 – -2.09	< 0.001	0.16
HSREpm	0.33 ± 0.10	0.46 ± 0.09	-0.11	-0.14 – -0.17	< 0.001	0.30

IND = individual high speed running threshold (60% vMAX); HSR = high speed running; HSRD = high speed running distance; HSR% = % of total distance at HSR; HSRE = number of HSR efforts; HSRDpm = high speed running distance per minute; HSREpm = high speed running efforts per minute.

With respect to differences between forwards and backs for the three dependent variables: total distance, relative distance and in-game maximum velocity, there was a statistically significant difference on the combined dependent variables, F (3, 186) = 40.91, p < 0.001, Wilk’s Lambda = 0.60, partial eta squared = 0.39. When the results of the dependent variables were considered separately, all variables reached statistical significance (Table 4).

**Table 4:** Forward vs back running game output.

Variable	Forward	Back	Mean Difference	95% CI of Mean Difference	p Value	Effect size (partial eta squared)
Total distance (m)	5638.64 ± 762.27	6171.85 ± 767.29	-733.21	-959.90 – -506.53	< 0.001	0.17
Relative distance (m·min^-1^)	71.61 ± 10.14	81.02 ± 10.20	-9.41	-12.43 – -6.39	< 0.001	0.16
In-game maximum velocity (m·s^-1)^	6.89 ± 0.61	7.94 ± 0.64	-1.05	-1.25 – -0.84	< 0.001	0.35

The differences in the ABS and IND HSR demands of each position sub-category in the forward unit are outlined in Table 5 with those of the back unit outlined in Table 6. Descriptive data relative to the inter-individual differences in HSR output within positional sub-categories are detailed in Tables 7 and 8.

**Table 5:** ABS vs IND HSR demands of forward position sub-categories.

Position	Variable	ABS	IND	Mean Difference	95% CI of Mean Difference
Prop	HSRD (m)	116.22 ± 74.25	375.96 ± 98.76*	-259.74	-307.45 – -212.02
	HSR% (%)	2.70 ± 2.20	8.37 ± 3.01*	-5.66	-7.11 – -4.22
	HSRE	8.18 ± 5.21	29.33 ± 9.42*	-21.14	-25.30 – -16.98
Hooker	HSRD (m)	267.40 ± 125.80	294.13 ± 83.24	-26.73	-106.51–53.05
	HSR% (%)	6.37 ± 3.90	6.88 ± 2.70	-0.51	-3.02–1.99
	HSRE	20.13 ± 10.62	21.26 ± 6.38	-1.13	-7.69–5.42
2^nd^ row	HSRD (m)	177.64 ± 71.85	338.57 ± 117.00*	-160.92	-212.95 – -108.90
	HSR% (%)	3.52 ± 1.52	6.75 ± 2.47*	-3.22	-4.35 – -2.12
	HSRE	13.03 ± 5.47	23.92 ± 9.81*	-10.89	-15.15 – -6.63
Number 8	HSRD (m)	447.20 ± 82.25	331.50 ± 92.60**	115.70	33.41–197.98
	HSR% (%)	7.90 ± 1.89	5.85 ± 1.80***	2.04	0.30–3.78
	HSRE	28.19 ± 7.66	19.20 ± 3.35**	9.20	3.64–14.75
Flanker	HSRD (m)	468.92 ± 117.59	395.52 ± 66.12**	73.40	19.14–127.65
	HSR% (%)	7.80 ± 2.11	6.58 ± 1.25***	1.22	0.23–2.20
	HSRE	32.16 ± 10.41	25.16 ± 4.63**	7.00	2.41–11.58

ABS = absolute high speed running threshold (5 m·s^-1^); IND = individual high speed running threshold (60% vMAX); HSR = high speed running; HSRD = high speed running distance; HSR% = % of total distance at HSR; HSRE = number of HSR efforts.

* = significantly different from ABS (p < 0.001);

** = significantly different from ABS (p < 0.01);

*** = significantly different from ABS (p < 0.05).

**Table 6:** ABS vs IND HSR demands of back position sub-categories.

Position	Variable	ABS	IND	Mean Difference	95% CI of Mean Difference
Outhalf	HSRD (m)	571.41 ± 102.49	433.16 ± 144.33***	138.25	32.26–244.23
	HSR% (%)	8.58 ± 1.02	6.58 ± 2.13**	1.99	0.57–3.40
	HSRE	38.25 ± 5.72	29.91 ± 6.35**	8.33	3.21–13.45
Scrumhalf	HSRD (m)	543.38 ± 232.80	527.61± 222.73	15.76	-168.60–200.19
	HSR% (%)	9.09 ± 3.29	8.86 ± 3.22	0.22	-2.41–2.86
	HSRE	34.15 ± 14.83	32.61 ± 12.79	1.53	-9.67–12.74
Centre	HSRD (m)	706.13 ± 208.63	587.00 ± 189.45	119.13	-2.11–240.38
	HSR% (%)	11.26 ± 3.31	9.51 ± 3.65	1.74	-0.37–3.86
	HSRE	45.18 ± 13.23	37.22 ± 10.69	7.95	-15.27 – -0.63
Wing	HSRD (m)	783.15 ± 145.19	639.96 ± 116.95*	143.19	69.75–216.63
	HSR% (%)	11.77 ± 1.84	9.67 ± 1.75*	2.09	1.09–3.09
	HSRE	42.38 ± 7.66	34.73 ± 6.65*	7.65	3.65–11.65
Fullback	HSRD (m)	784.00 ± 168.33	570.15 ± 125.18**	213.84	93.76–333.93
	HSR% (%)	12.20 ± 2.37	8.82 ± 1.56***	3.37	1.75–5.00
	HSRE	44.23 ± 10.74	35.84 ± 8.47*	8.35	0.55–16.21

ABS = absolute high speed running threshold (5 m·s^-1^); IND = individual high speed running threshold (60% vMAX); HSR = high speed running; HSRD = high speed running distance; HSR% = % of total distance at HSR; HSRE = number of HSR efforts.

* = significantly different from ABS (p < 0.001);

** = significantly different from ABS (p < 0.01);

*** = significantly different from ABS (p < 0.05).

**Table 7:** Descriptive statistics on the IND HSR output of individual forwards players.

	Total distance (m)	Relative distance (m·min^-1^)	In-game maximum velocity (m·s^-1^)	HSRD (m)	HSR% (%)	HSRE	HSRDpm (m·min^-1^)	HSREpm
Prop [1]	5136.50	67.59	6.23	294.50	3.86	5.70	25.75	0.34
Prop [2]	4478.33	64.26	6.48	372.830	5.35	8.34	29.00	0.41
Prop [3]	4729.80	72.35	6.01	378.70	5.89	8.18	26.90	0.42
Prop [4]	4073.00	59.89	6.10	455.75	6.97	12.06	41.50	0.60
Hooker [1]	4546.83	63.89	7.03	277.160	3.96	6.48	20.16	0.28
Hooker [2]	5245.00	73.00	6.10	289.00	4.14	5.69	21.33	0.30
Hooker [3]	3429.33	51.83	6.05	338.33	5.10	10.07	23.33	0.35
2^nd^ Row [1]	5410.00	71.55	6.24	316.16	4.146	5.85	25.00	0.33
2^nd^ Row [2]	5268.58	72.33	6.73	401.50	5.52	7.69	27.16	0.37
2^nd^ Row [3]	4289.66	64.05	6.56	270.66	4.39	6.83	20.00	0.29
2^nd^ Row [4]	5565.20	70.39	6.49	280.60	3.54	4.96	22.40	0.28
Number 8 [1]	5887.37	73.59	7.56	328.25	4.10	5.60	20.50	0.25
Number 8 [2]	5366.33	69.00	7.04	383.00	4.96	7.22	20.33	0.26
Flanker [1]	6325.60	79.07	7.45	408.20	5.10	6.47	27.20	0.34
Flanker [2]	6130.33	81.33	8.33	435.33	5.72	7.12	28.00	0.36
Flanker [3]	5933.00	74.16	7.73	410.85	5.13	6.96	26.85	0.33
Flanker [4]	6435.28	81.66	7.37	356.57	4.52	5.58	20.57	0.26

IND = individual high speed running threshold (60% vMAX); HSR = high speed running; HSRD = high speed running distance; HSR% = % of total distance at HSR; HSRE = number of HSR efforts; HSRDpm = high speed running distance per minute; HSREpm = high speed running efforts per minute.

**Table 8:** Descriptive statistics on the IND HSR output of individual backs players.

	Total distance (m)	Relative distance (m·min^-1^)	In-game maximum velocity (m·s^-1^)	HSRD (m)	HSR% (%)	HSRE	HSRDpm (m·min^-1^)	HSREpm
Outhalf [1]	7053.66	88.17	8.42	462.00	5.77	6.65	32.50	0.40
Outhalf [2]	6287.16	78.58	7.66	404.33	5.05	6.52	27.33	0.34
Scrumhalf [1]	6328.83	83.31	7.50	433.83	5.65	6.79	28.50	0.37
Scrumhalf [2]	5392.33	78.26	7.633	492.00	7.28	9.49	28.33	0.41
Centre [1]	6581.66	82.55	7.71	467.50	5.85	7.10	32.50	0.40
Centre [2]	5751.66	71.89	8.24	605.16	7.56	11.32	34.83	0.43
Centre [3]	6309.00	78.86	8.22	596.00	7.45	9.24	37.00	0.46
Centre [4]	6700.83	83.76	8.01	682.33	8.52	10.29	44.50	0.55
Wing [1]	6543.25	82.65	8.14	611.87	7.71	9.37	33.25	0.41
Wing [2]	6827.57	86.01	8.31	579.57	7.30	8.57	32.00	0.40
Wing [3]	6590.60	82.38	8.41	662.20	8.27	10.10	35.40	0.44
Wing [4]	6700.00	83.75	8.33	730.00	9.12	10.89	36.33	0.45
Wing [5]	6585.25	82.31	8.50	693.25	8.66	10.62	40.00	0.50
Fullback [1]	6501.57	82.37	7.97	590.14	7.45	9.024	35.28	0.44
Fullback [2]	6163.00	77.03	8.00	534.00	6.67	8.68	35.80	0.44

IND = individual high speed running threshold (60% vMAX); HSR = high speed running; HSRD = high speed running distance; HSR% = % of total distance at HSR; HSRE = number of HSR efforts; HSRDpm = high speed running distance per minute; HSREpm = high speed running efforts per minute.

## Discussion

The results confirm the main hypothesis of the study. individualisation of the HSR threshold to 60% of individual Vmax reveals a significant underestimation of HSR requirements for forwards and a significant overestimation of HSR requirements for backs generated by use of a standardised HSR threshold of 5m·s^-1^ (Table 1). Furthermore, individuals and position sub-categories whose Vmax varies greatly from the group mean (i.e. forward or back mean) have the largest over- or underestimation of HSR demands under the condition of application of an absolute HSR threshold. The findings of the current study indicated that the largest over-estimation of HSR demands is for the full-back, wing and outhalf position sub-categories, while the largest under-estimation is for the prop and second row positions sub-categories. To a lesser degree the HSR demands of the number 8 and flanker position sub-categories are overestimated. The hooker, scrum-half and centre position sub-categories are not significantly different when an individualised or absolute HSR threshold is applied.

The current study demonstrates that if HSR is considered to have an absolute threshold of 5m·s^-1^ (18km·h^-1^), backs have a much greater requirement for HSR in all measured metrics (HSRD, HSR%, HSRE, HSRDpm, HSREpm) when compared to forwards (Table 2). Reporting HSR demands with absolute zones is not without merit as it allows comparison of performance between individuals using a standardised measure. Research has reported that across rugby football codes, higher level performers tend to be faster with higher levels of competition involving greater HSR demands [8,9,12]. Therefore, absolute HSR performance may relate to an individual players ability to compete at an elite level. However, this method of analysis is limited in its applicability to informing training prescription as it fails to account for the large variance in Vmax between position sub-categories and inter-individually within these position sub-categories. Additionally, considering that absolute HSR performance is not interpreted relative to the Vmax capability of the individual player, it fails to assist in informing the practitioner of the individual’s relative training or playing load. The results of the present study illustrate the limitation of prescribing training based on an absolute threshold for HSR, which will likely result in under prescription for forwards and over prescription for backs, with the degree of error fluctuating between position sub-categories and on a player to player basis.

When considering the use of an absolute in comparison to an individualised HSR threshold, there was a significant underestimation of HSR distance (HSRD), HSR% and HSR efforts (HSRE) for forwards (Table 1). In contrast, there was a significant overestimation of the same HSR metrics for backs (Table 1) with the use of an absolute threshold. In the absolute HSR threshold condition, HSRD for forwards was 269 ± 172m in comparison to 698 ± 198m for backs. This suggests that backs complete approximately 2.6 times the HSR distance of forwards. However in the individualised HSR threshold condition, HSR distance for forwards was 355 ± 99m and HSR distance for backs was 570 ± 171m. This suggests that HSR requirement for backs are 1.6 times that of forwards. Similarly, HSR% under the absolute condition is 5.15 ± 3.2% for forwards and 10.85 ± 2.8% for backs (approximately 2.1 times greater for backs in comparison to forwards). In the individualised condition HSR% for forwards is reported as 7.1 ± 2.5% for forwards and 8.9 ± 2.8% for backs (approximately 1.25 times greater for backs in comparison to forwards). HSRE in the absolute condition is reported as 18.8 ± 12 for forwards and 41.6±11 for backs (approximately 2.2 times greater requirements for backs in comparison to forwards). In the individualised condition HSRE is reported as 24.8 ± 8 for forwards and 34.5±9 for backs (approximately 1.4 times greater for backs than for forwards). This represents a significant shift in the interpretation of HSR demands of the sport between the two major positional groups depending on the type of HSR threshold applied. In contrast to the application of an absolute HSR threshold, when individual Vmax capabilities are considered, the HSR demands of forwards are much closer to those of backs.

When forwards are divided into the five discernible position sub-categories, the differential between HSR demands derived from individualised versus absolute thresholds varies greatly (Table 5). Props were shown to have the greatest difference, having more than three times the HSRD, HSR% and HSRE in the individualised condition versus the absolute. Second row players had roughly double the HSR demands across all HSR metrics in the individual condition. As a positional sub-category, hookers showed no significant changes in HSR demands between measurement conditions, while the number 8 and flanker position sub-categories where characterized by a 15–25% decrease in HSR demands across all metrics in the individualised measurement condition in comparison to the absolute.

A similar trend is observed in an analysis of HSR demands of the five backs position sub-categories (Table 6). The outhalf, wing and fullback position sub-categories all exhibited a decrease in HSR demands under the condition of an individualised HSR threshold. Although these decreases are statistically significant, the magnitude of the change is not as large (18–28% across all HSR metrics) as that observed in the prop and second row position sub-categories. This study reports no statistically significant changes in HSR metrics for the centre and scrumhalf position sub-categories in the individualised versus absolute condition.

There is also large variability in Vmax capabilities of some players within the same position sub-categories (Tables 7 & 8). When considering the hooker position in the current study, hooker 1 had a Vmax of 9.3 m·s^-1^ compared to hookers 2 and 3 who had a Vmax of 7.7m·s^-1^ and 7.8m·s^-1^ respectively. As a result, considering the HSR demands of this position sub-category based of the mean Vmax of the group (8.3m·s^-1^ in this case) will likely result in over or underestimation of HSR demands for each individual. The HSR demands must therefore be considered, not only between, but also within position sub-categories according to each player’s Vmax.

Using GPS technology and a large data sample, the current study has the potential to add significantly to an understanding of the HSR demands of the game by practitioners. Existing research in the area is limited by less accurate GPS technology [3,9,11,12,16]. Much of the existing research also uses participants of sub-elite standing [12] or a small sample size [3,11,12]. The only other study to use a comparably large sample of GPS data [9] used an individualised HSR threshold of 51% Vmax. It is questionable as to whether a player can be considered to be moving at high speed at 51% of his capacity. The aforementioned study also considered Vmax to be in-game Vmax. The current study indicates that- even over the course of a league campaign, very few players achieve true Vmax during game play. Basing HSR threshold on an individual percentage of a Vmax value that is likely to be less than true Vmax may lead to over reporting of HSR metrics. This may account for the differences between the aforementioned and present study in reporting of how much of total distance is covered at high speed between position groups.

It is important to note that an analysis of HSR demands alone does not provide a comprehensive profile of the locomotive demands of rugby union. Given the short distances over which running efforts occur [19,20,21], the acceleration demands of the game are likely to be a key component of game-play. Although research has shown GPS to be a valid and reliable measure of accelerations in a controlled environment, [22] our data suggests that further investigation and adaptations in data processing are required to attain ecological validity in detecting accelerations using GPS technology.

This study does not account for the collision component of the game and the high intensity static exertions which are well documented and considered as being crucial, particularly for forwards positions [5,7,8]. GPS collision detection with catapult technology has been validated for rugby league but at present, not for rugby union [23]. With these limitations in mind, and with recognition of current technological capabilities, it may be that, in the short-term, video analysis of collisions combined with GPS based locomotor profiles is the path to building a more complete picture of the game demands of rugby union.

This study has merit in comparing the effect of individualised versus global thresholds on the interpretation of the running demands of the game. To that end, it is necessary to compare the HSR output of the same players under the two conditions. Existing studies that use data compared across a number of teams and use a wider subject group may provide a better source of comparison between teams.

In conclusion the findings of the current study suggest that previous reports may underestimate the HSR demands of position sub-categories such as the prop and 2^nd^ row due to their lower Vmax values. Furthermore, players’ with Vmax values that constitute an anomaly within their position sub-category may also be misrepresented if demands are considered relative to the group average and not individualized. Therefore, practitioners should consider carefully the HSR demands of each individual player in prescribing appropriate training for the sport. The results of the present study underscore the need to individualise speed zones and to consider players on an individual basis to accurately gauge the HSR demands of rugby union.

## Supporting Information

S1 Dataset Compiled data for all reported metrics; Absolute & Individualised thresholds. (XLSX)
